# Exploring knowledge and attitudes towards dementia risk prediction, barriers to dementia services and service improvement recommendations with diverse populations in the UK

**DOI:** 10.1002/alz70860_101011

**Published:** 2025-12-23

**Authors:** Rupinder Kaur Bajwa, Matilda Hanjari, Amani Al‐Oraibi, Ralph Kwame Akeya, Manjot Brar, Louise Robinson, Blossom CM Stephan, Nadeem Qureshi, Manpreet Bains

**Affiliations:** ^1^ University of Nottingham, Nottingham, Nottinghamshire, United Kingdom; ^2^ Newcastle University, Newcastle, Newcastle, United Kingdom; ^3^ NIHR ARC North East and North Cumbria, Newcastle, Newcastle, United Kingdom; ^4^ Curtin University, Perth, Western Australia, Australia

## Abstract

**Background:**

In the UK there is no recommended method for predicting dementia risk and current prediction models available have been developed using homogenous datasets. This is concerning given that low‐income and ethnic minority groups are at high risk for dementia and face inequalities in accessing early diagnosis and treatment.

**Method:**

We conducted a qualitative study using task group methodology to explore knowledge of dementia, attitudes towards dementia risk prediction, and barriers and facilitators to accessing dementia services for diverse populations in England. Participants were recruited through convenience and purposeful sampling and task groups were held at local community group venues or online. Data were analysed using thematic analysis.

**Results:**

One hundred and forty‐nine participants aged between 40 and 80, representing low‐income and ethnically diverse groups participated in the task groups. Participants had some knowledge about dementia but there were challenges accessing information. Participants also shared concerns about healthcare professionals' lack of understanding of dementia. Knowledge of risk factors was comprehensive but limited for dementia risk prediction and screening. Attitudes towards risk prediction were positive and using risk prediction tools was viewed as empowering. Participants stressed the need to improve awareness and consider the potential psychological impact of a high‐risk result. Barriers to dementia services for diverse groups included challenges in accessing healthcare services, stigma, language, and cultural/religious views about dementia. Recommendations for service improvement include workforce training on cultural awareness, improving language support in dementia services and tailored community engagement approaches to improve understanding of dementia risk prediction.

**Conclusion:**

This study provides insights into current knowledge and attitudes towards dementia, risk prediction and the use of risk prediction tools among diverse populations in England. Barriers to health services for diverse groups alongside service improvement recommendations provide a starting point for health services to develop culturally aware and inclusive dementia services.

